# 2′,3,4,4′-Tetra­meth­oxy­chalcone

**DOI:** 10.1107/S1600536810022142

**Published:** 2010-06-26

**Authors:** Johannes H. van Tonder, Theunis J. Muller, Barend C. B. Bezuidenhoudt

**Affiliations:** a Department of Chemistry, University of the Free State, PO Box 339, Bloemfontein, 9300, South Africa

## Abstract

In the title compound [systematic name: 1-(2,4-dimethoxyphenyl)-3-(3,4-dimethoxyphenyl)prop-2-en-1-one], C_19_H_20_O_5_, the dihedral angle between the benzene rings is 26.88 (5)°. One of the meth­oxy groups is twisted slightly away from the plane [C—O—C—C torsion angle = −12.8 (3)°] while the others are almost co-planer [C—O—C—C torsion angles = −3.2 (3), 2.6 (3) and −3.6 (3)°]. The crystal packing is stabilized by inter­molecular C—H⋯O inter­actions. A weak intra­molecular C—H⋯O inter­action occurs.

## Related literature

For properties and uses of chalcones, see: Marais *et al.* (2005 ▶); Fichou *et al.* (1988 ▶); Uchida *et al.* (1998 ▶). For the biological activity of flavenoids, see: Pietta *et al.* (2003 ▶). For related structures, see: Patil *et al.* (2006**a* ▶,*b* ▶,c* ▶); Teh *et al.* (2006*a* ▶,*b* ▶,*c* ▶); Rosli *et al.* (2006 ▶). For the synthesis of the title compound, see: Kraus *et al.* (2008 ▶). For bond-length data, see: Allen *et al.* (1987 ▶).
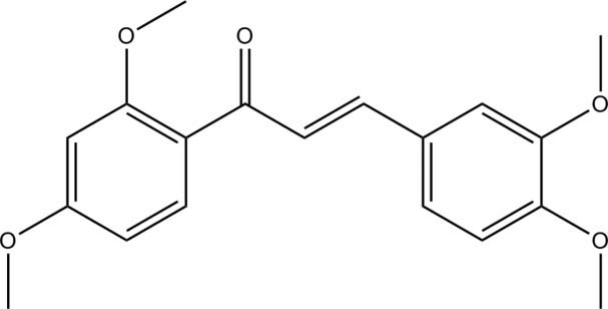


## Experimental

### 

#### Crystal data

C_19_H_20_O_5_
*M* *_r_* = 328.35
Monoclinic, 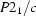

*a* = 12.5839 (7) Å
*b* = 11.7204 (7) Å
*c* = 12.1339 (6) Å
β = 109.489 (2)°
*V* = 1687.07 (16) Å^3^
*Z* = 4
Mo *K*α radiation
μ = 0.09 mm^−1^
*T* = 100 K
0.49 × 0.22 × 0.07 mm

#### Data collection

Bruker APEXII diffractometer
Absorption correction: multi-scan (*SADABS*; Bruker, 2008 ▶) *T*_min_ = 0.976, *T*_max_ = 0.994
32681 measured reflections
4205 independent reflections
2539 reflections with *I* > 2σ(*I*)
*R*_int_ = 0.039

#### Refinement

*R*[*F* ^2^ > 2σ(*F* ^2^)] = 0.044
*wR*(*F* ^2^) = 0.188
*S* = 1.12
4205 reflections
225 parameters
H atoms treated by a mixture of independent and constrained refinement
Δρ_max_ = 0.21 e Å^−3^
Δρ_min_ = −0.20 e Å^−3^

### 

Data collection: *APEX2* (Bruker, 2008 ▶); cell refinement: *SAINT-Plus* (Bruker, 2008 ▶); data reduction: *SAINT-Plus* and *XPREP* (Bruker, 2008 ▶); program(s) used to solve structure: *SHELXS97* (Sheldrick, 2008 ▶); program(s) used to refine structure: *SHELXL97* (Sheldrick, 2008 ▶); molecular graphics: *DIAMOND* (Brandenberg & Putz, 2005 ▶); software used to prepare material for publication: WingGX (Farrugia, 1999 ▶).

## Supplementary Material

Crystal structure: contains datablocks global, I. DOI: 10.1107/S1600536810022142/hg2688sup1.cif

Structure factors: contains datablocks I. DOI: 10.1107/S1600536810022142/hg2688Isup2.hkl

Additional supplementary materials:  crystallographic information; 3D view; checkCIF report

## Figures and Tables

**Table 1:** Hydrogen-bond geometry (Å, °)

*D*—H⋯*A*	*D*—H	H⋯*A*	*D*⋯*A*	*D*—H⋯*A*
C8—H8⋯O1	0.96 (2)	2.29 (2)	2.813 (3)	113.6 (15)
C18—H18*B*⋯O3^i^	0.96	2.46	3.253 (3)	140

Symmetry code: (i) 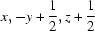.

**Fig. 1.**
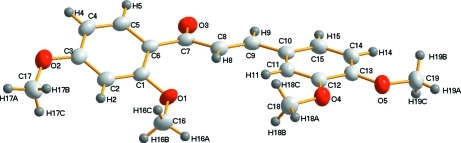
Representation of the title compound, showing the numbering scheme and displacement ellipsoids (50% probability).

## supplementary crystallographic information

### Comment

Flavonoids are a prominent class of secondary plant metabolites known for their wide range of biological active compounds that exibit antibacterial, antifugal, antitumor and anti-inflammatory properties (Pietta *et al.*, 2003). Chalcones are an important subclass of these compounds and are often utilized as intermediates in the synthesis of a variety of cyclic flavonoids (Marais *et al.*, 2005). Furthermore, many chalcone derivatives are known to have excellent non-linear optical (NLO) properties (Fichou *et al.*, 1988; Uchida *et al.*, 1998; Patil *et al.*, 2006*a*,b). We report here a new chalcone which we have successfully synthesized (the title compound, (I)). Bond distances in (I) have normal values (Allen *et al.*, 1987) and bond angles and distances are comparable to those in related structures (Teh *et al.*, 2006*a*,b,c; Patil *et al.*, 2006*a*,b,c; Rosli *et al.* 2006). The least squares plane through the enone group (C7, C8, C9 and O3) exhibit dihedral angles of 29.2 (1)° and 4.5 (1)° with the C1—C6 and C10—C15 benzene rings, respectively. The dihedral angle between the two benzene rings is 26.88 (5)°. The methoxy group attached at C1 is slightly twisted away from the C1—C6 benzene ring plane, with a C16—O1—C1—C2 torsion angle of -12.8 (3)°. The methoxy groups at C3, C12 and C13 are almost co-planar with the C1—C6 and C10—C15 benzene rings with C17—O2—C3—C2, C18—O4—C12—C11 and C19—O5—C13—C14 torsion angels of -3.2 (3)°, 2.6 (3)° and -3.6 (3)°, respectively. An intramolecular C8—H8···O1 hydrogen bond is observed in the molecular structure of (I), while the molecules form chains through intermolecular C18—H18B···O3^i^ hydrogen bonds (Table 1).

### Experimental

The title compoud was synthesized according to the procedure by Kraus *et al.* (2008) Freshly ground KOH (1.55 g; 27.80 mmol; 5 eq.) was added to a cold (ice bath) stirring solution of 2',4'-dimethoxyacetophenone (1.00 g; 5.56 mmol) and 3,4-dimethoxybenzaldehyde (1.13 g; 7.12 mmol; 1.2 eq.) in EtOH (40 ml). The reaction mixture was allowed to heat to room temperature and stirred to completion (TLC). Ice was added to the reaction mixture prior to acidification with concentrated HCl (litmus). Extraction was performed with EtOAc (3 *x* 100 ml) and the organic fractions combined. The organic phase was neutralized with a saturated solution of NaHCO_3_ (litmus), washed with water, dried (Na_2_SO_4_) and evaporated *in vacuo* at *ca* 40 °C. Crystallization from EtOH afforded the desired chalcone (1.55 g; 84.7%) as yellow needles. *R_f_* 0.16 (H:A; 8:2); Mp 88.3 °C; ^1^H NMR (600 MHz, CDCl_3_) δ 7.69 (1*H*, d, *J* = 8.61 Hz, H-6'), 7.58 (1*H*, d, *J* = 15.71 Hz, H-β), 7.33 (1*H*, d, *J* = 15.71 Hz, H-α), 7.14 (1*H*, dd, *J* = 1.92, 8.32 Hz, H-5), 7.08 (1*H*, d, *J* = 1.92 Hz, H-2), 6.84 (1*H*, d, *J* = 8.32 Hz, H-6), 6.52 (1*H*, dd, *J* = 2.25, 8.61 Hz, H-5'), 6.46 (1*H*, d, *J* = 2.25 Hz, H– 3'), 3.88 (3*H*, s, –OCH_3_), 3.87 (3*H*, s, –OCH_3_), 3.85 (3*H*, s, –OCH_3_), 3.82 (3*H*, s, –OCH_3_); ^13^C NMR (151 MHz, CDCl_3_) δ 190.58, 163.96, 160.21, 150.95, 149.11, 142.34 (C-β), 132.63 (C-6'), 128.39, 125.28 (C-α), 122.60 (C-5), 122.36, 111.13 (C-6), 110.24 (C-2), 105.14 (C-5'), 98.66 (C-3'), 55.94 (–OCH_3_), 55.86 (–OCH_3_), 55.71 (–OCH_3_), 55.50 (–OCH_3_).

### Refinement

The aromatic H atoms were placed in geometrically idealized positions and constrained to ride on its parent atoms with *U*_iso_ (H) = 1.2*U*_eq_(C) and at a distance of 0.93 Å. The hydrogen atoms of the methine (H8 and H9) group were determined from a difference Fourier map and their positional parameters freely refined (C8—H8 = 0.96 (2)Å and C9—H9 = 1.01 (2) Å). The methyl H atoms were placed in geometrically idealized positions and constrained to ride on its parent atoms with *U*_iso_(H) = 1.5*U*_eq_(C) and at a distance of 0.96 Å.

### Crystal data

**Table:**

C_19_H_20_O_5_	*F*(000) = 696
*M**_r_* = 328.35	*D*_x_ = 1.293 Mg m^−^^3^
Monoclinic, *P*2_1_/*c*	Mo *K*α radiation, λ = 0.71073 Å
Hall symbol: -P 2ybc	Cell parameters from 7154 reflections
*a* = 12.5839 (7) Å	θ = 2.4–23.6°
*b* = 11.7204 (7) Å	µ = 0.09 mm^−^^1^
*c* = 12.1339 (6) Å	*T* = 100 K
β = 109.489 (2)°	Plate, colourless
*V* = 1687.07 (16) Å^3^	0.49 × 0.22 × 0.07 mm
*Z* = 4	

### Data collection

**Table:**

Bruker APEXII diffractometer	2539 reflections with *I* > 2σ(*I*)
graphite	*R*_int_ = 0.039
phi and ω scans	θ_max_ = 28.4°, θ_min_ = 1.7°
Absorption correction: multi-scan (*SADABS*; Bruker, 2008)	*h* = −16→16
*T*_min_ = 0.976, *T*_max_ = 0.994	*k* = −15→15
32681 measured reflections	*l* = −11→16
4205 independent reflections	

### Refinement

**Table:**

Refinement on *F*^2^	Primary atom site location: structure-invariant direct methods
Least-squares matrix: full	Secondary atom site location: difference Fourier map
*R*[*F*^2^ > 2σ(*F*^2^)] = 0.044	Hydrogen site location: inferred from neighbouring sites
*wR*(*F*^2^) = 0.188	H atoms treated by a mixture of independent and constrained refinement
*S* = 1.12	*w* = 1/[σ^2^(*F*_o_^2^) + (0.0998*P*)^2^ + 0.0628*P*] where *P* = (*F*_o_^2^ + 2*F*_c_^2^)/3
4205 reflections	(Δ/σ)_max_ = 0.001
225 parameters	Δρ_max_ = 0.21 e Å^−^^3^
0 restraints	Δρ_min_ = −0.20 e Å^−^^3^

### Special details

**Table:**

Geometry. All s.u.'s (except the s.u. in the dihedral angle between two l.s. planes) are estimated using the full covariance matrix. The cell s.u.'s are taken into account individually in the estimation of s.u.'s in distances, angles and torsion angles; correlations between s.u.'s in cell parameters are only used when they are defined by crystal symmetry. An approximate (isotropic) treatment of cell s.u.'s is used for estimating s.u.'s involving l.s. planes.
Refinement. Refinement of *F*^2^ against ALL reflections. The weighted *R*-factor *wR* and goodness of fit *S* are based on *F*^2^, conventional *R*-factors *R* are based on *F*, with *F* set to zero for negative *F*^2^. The threshold expression of *F*^2^ > 2σ(*F*^2^) is used only for calculating *R*-factors(gt) *etc*. and is not relevant to the choice of reflections for refinement. *R*-factors based on *F*^2^ are statistically about twice as large as those based on *F*, and *R*- factors based on ALL data will be even larger.

### Fractional atomic coordinates and isotropic or equivalent isotropic displacement parameters (Å^2^)

**Table:**

	*x*	*y*	*z*	*U*_iso_*/*U*_eq_	
C1	0.57536 (15)	0.12721 (14)	0.10767 (13)	0.0572 (4)	
C2	0.66866 (16)	0.07753 (15)	0.09002 (15)	0.0635 (5)	
H2	0.7138	0.027	0.1449	0.076*	
C3	0.69406 (17)	0.10359 (16)	−0.00938 (16)	0.0665 (5)	
C4	0.62632 (18)	0.17787 (18)	−0.09154 (16)	0.0724 (5)	
H4	0.6422	0.1941	−0.1594	0.087*	
C5	0.53595 (17)	0.22720 (17)	−0.07222 (14)	0.0654 (5)	
H5	0.4918	0.2781	−0.1274	0.079*	
C6	0.50707 (15)	0.20426 (14)	0.02731 (13)	0.0555 (4)	
C7	0.40909 (15)	0.26733 (15)	0.04011 (13)	0.0582 (4)	
C8	0.33980 (16)	0.21568 (16)	0.10368 (15)	0.0603 (5)	
C9	0.25176 (15)	0.26831 (16)	0.11757 (14)	0.0596 (5)	
C10	0.18227 (14)	0.22631 (15)	0.18413 (14)	0.0570 (4)	
C11	0.20392 (14)	0.12066 (15)	0.24240 (14)	0.0571 (4)	
H11	0.2627	0.0756	0.2367	0.068*	
C12	0.14066 (14)	0.08267 (15)	0.30728 (14)	0.0574 (4)	
C13	0.05259 (14)	0.15026 (17)	0.31711 (15)	0.0614 (5)	
C14	0.02979 (16)	0.25345 (18)	0.26000 (16)	0.0692 (5)	
H14	−0.0292	0.2982	0.2656	0.083*	
C15	0.09404 (16)	0.29117 (17)	0.19425 (16)	0.0676 (5)	
H15	0.0777	0.3612	0.1563	0.081*	
C16	0.60374 (18)	0.01862 (18)	0.28377 (17)	0.0782 (6)	
H16A	0.5762	0.0164	0.3486	0.117*	
H16B	0.6836	0.0318	0.312	0.117*	
H16C	0.5882	−0.0529	0.2429	0.117*	
C17	0.86153 (19)	−0.0100 (2)	0.0519 (2)	0.0954 (7)	
H17A	0.9212	−0.0335	0.0241	0.143*	
H17B	0.8224	−0.0761	0.0653	0.143*	
H17C	0.8927	0.0315	0.1237	0.143*	
C18	0.24973 (17)	−0.08614 (17)	0.36379 (18)	0.0710 (5)	
H18A	0.2528	−0.1544	0.4085	0.107*	
H18B	0.3184	−0.0439	0.3967	0.107*	
H18C	0.2405	−0.1061	0.2844	0.107*	
C19	−0.09188 (18)	0.1716 (2)	0.4026 (2)	0.0927 (7)	
H19A	−0.1252	0.1302	0.451	0.139*	
H19B	−0.1484	0.1879	0.3286	0.139*	
H19C	−0.0607	0.2418	0.4403	0.139*	
O1	0.54938 (13)	0.10796 (12)	0.20672 (10)	0.0780 (4)	
O2	0.78452 (13)	0.06147 (14)	−0.03354 (13)	0.0874 (5)	
O3	0.38578 (12)	0.36122 (11)	−0.00560 (11)	0.0767 (4)	
O4	0.15725 (11)	−0.01826 (11)	0.36644 (12)	0.0717 (4)	
O5	−0.00515 (11)	0.10484 (13)	0.38441 (12)	0.0785 (4)	
H8	0.3591 (17)	0.1401 (19)	0.1332 (17)	0.075 (6)*	
H9	0.2294 (16)	0.3460 (17)	0.0808 (16)	0.071 (5)*	

### Atomic displacement parameters (Å^2^)

**Table:**

	*U*^11^	*U*^22^	*U*^33^	*U*^12^	*U*^13^	*U*^23^
C1	0.0751 (11)	0.0504 (9)	0.0446 (8)	−0.0050 (8)	0.0182 (8)	−0.0034 (7)
C2	0.0785 (12)	0.0527 (10)	0.0560 (9)	−0.0032 (9)	0.0180 (9)	−0.0043 (8)
C3	0.0762 (12)	0.0620 (12)	0.0645 (10)	−0.0128 (9)	0.0279 (9)	−0.0147 (9)
C4	0.0920 (14)	0.0757 (13)	0.0567 (10)	−0.0123 (11)	0.0342 (10)	−0.0027 (9)
C5	0.0802 (13)	0.0633 (11)	0.0498 (9)	−0.0111 (9)	0.0176 (8)	0.0044 (8)
C6	0.0685 (10)	0.0508 (9)	0.0431 (8)	−0.0099 (8)	0.0132 (7)	−0.0021 (7)
C7	0.0725 (11)	0.0511 (10)	0.0446 (8)	−0.0058 (8)	0.0110 (7)	0.0012 (7)
C8	0.0714 (12)	0.0497 (10)	0.0566 (9)	−0.0034 (9)	0.0171 (8)	0.0030 (8)
C9	0.0670 (11)	0.0513 (10)	0.0526 (9)	−0.0035 (9)	0.0094 (8)	−0.0001 (8)
C10	0.0586 (10)	0.0538 (10)	0.0518 (8)	−0.0006 (8)	0.0092 (7)	−0.0036 (7)
C11	0.0563 (9)	0.0538 (10)	0.0592 (9)	0.0007 (8)	0.0167 (8)	−0.0018 (8)
C12	0.0565 (10)	0.0527 (10)	0.0593 (9)	−0.0041 (8)	0.0143 (8)	−0.0027 (8)
C13	0.0541 (9)	0.0673 (12)	0.0610 (9)	−0.0043 (8)	0.0168 (8)	−0.0096 (9)
C14	0.0586 (11)	0.0715 (13)	0.0739 (11)	0.0105 (9)	0.0172 (9)	−0.0051 (10)
C15	0.0668 (11)	0.0596 (11)	0.0677 (11)	0.0070 (9)	0.0110 (9)	0.0027 (9)
C16	0.0932 (14)	0.0743 (13)	0.0629 (10)	0.0076 (11)	0.0204 (10)	0.0213 (10)
C17	0.0753 (14)	0.1100 (19)	0.0954 (16)	0.0027 (13)	0.0213 (12)	−0.0291 (14)
C18	0.0812 (13)	0.0533 (11)	0.0818 (12)	0.0052 (9)	0.0316 (10)	0.0076 (9)
C19	0.0673 (12)	0.125 (2)	0.0908 (14)	0.0056 (13)	0.0336 (11)	−0.0140 (14)
O1	0.1039 (10)	0.0834 (10)	0.0517 (7)	0.0266 (8)	0.0326 (7)	0.0199 (6)
O2	0.0914 (10)	0.0909 (11)	0.0904 (10)	−0.0003 (8)	0.0444 (8)	−0.0111 (8)
O3	0.0972 (10)	0.0607 (8)	0.0724 (8)	0.0086 (7)	0.0284 (7)	0.0193 (7)
O4	0.0769 (9)	0.0598 (8)	0.0869 (9)	0.0028 (6)	0.0385 (7)	0.0107 (6)
O5	0.0707 (8)	0.0858 (10)	0.0877 (9)	−0.0012 (7)	0.0378 (7)	−0.0051 (7)

### Geometric parameters (Å, °)

**Table:**

C1—O1	1.3654 (19)	C12—C13	1.399 (2)
C1—C2	1.390 (3)	C13—O5	1.369 (2)
C1—C6	1.395 (2)	C13—C14	1.376 (3)
C2—C3	1.381 (3)	C14—C15	1.384 (3)
C2—H2	0.93	C14—H14	0.93
C3—O2	1.359 (2)	C15—H15	0.93
C3—C4	1.382 (3)	C16—O1	1.418 (2)
C4—C5	1.364 (3)	C16—H16A	0.96
C4—H4	0.93	C16—H16B	0.96
C5—C6	1.398 (2)	C16—H16C	0.96
C5—H5	0.93	C17—O2	1.430 (3)
C6—C7	1.489 (3)	C17—H17A	0.96
C7—O3	1.223 (2)	C17—H17B	0.96
C7—C8	1.473 (3)	C17—H17C	0.96
C8—C9	1.327 (3)	C18—O4	1.419 (2)
C8—H8	0.96 (2)	C18—H18A	0.96
C9—C10	1.460 (3)	C18—H18B	0.96
C9—H9	1.01 (2)	C18—H18C	0.96
C10—C15	1.384 (3)	C19—O5	1.418 (3)
C10—C11	1.407 (2)	C19—H19A	0.96
C11—C12	1.367 (2)	C19—H19B	0.96
C11—H11	0.93	C19—H19C	0.96
C12—O4	1.363 (2)		
			
O1—C1—C2	121.95 (15)	O5—C13—C12	115.08 (17)
O1—C1—C6	116.73 (16)	C14—C13—C12	119.51 (17)
C2—C1—C6	121.24 (15)	C13—C14—C15	120.49 (17)
C3—C2—C1	119.70 (17)	C13—C14—H14	119.8
C3—C2—H2	120.1	C15—C14—H14	119.8
C1—C2—H2	120.1	C14—C15—C10	121.04 (18)
O2—C3—C2	124.22 (19)	C14—C15—H15	119.5
O2—C3—C4	115.62 (17)	C10—C15—H15	119.5
C2—C3—C4	120.16 (18)	O1—C16—H16A	109.5
C5—C4—C3	119.46 (17)	O1—C16—H16B	109.5
C5—C4—H4	120.3	H16A—C16—H16B	109.5
C3—C4—H4	120.3	O1—C16—H16C	109.5
C4—C5—C6	122.60 (18)	H16A—C16—H16C	109.5
C4—C5—H5	118.7	H16B—C16—H16C	109.5
C6—C5—H5	118.7	O2—C17—H17A	109.5
C1—C6—C5	116.82 (17)	O2—C17—H17B	109.5
C1—C6—C7	125.93 (15)	H17A—C17—H17B	109.5
C5—C6—C7	117.21 (15)	O2—C17—H17C	109.5
O3—C7—C8	120.85 (17)	H17A—C17—H17C	109.5
O3—C7—C6	118.74 (16)	H17B—C17—H17C	109.5
C8—C7—C6	120.39 (15)	O4—C18—H18A	109.5
C9—C8—C7	122.73 (18)	O4—C18—H18B	109.5
C9—C8—H8	120.1 (12)	H18A—C18—H18B	109.5
C7—C8—H8	117.1 (12)	O4—C18—H18C	109.5
C8—C9—C10	126.39 (18)	H18A—C18—H18C	109.5
C8—C9—H9	118.9 (11)	H18B—C18—H18C	109.5
C10—C9—H9	114.7 (11)	O5—C19—H19A	109.5
C15—C10—C11	117.73 (17)	O5—C19—H19B	109.5
C15—C10—C9	120.67 (17)	H19A—C19—H19B	109.5
C11—C10—C9	121.57 (16)	O5—C19—H19C	109.5
C12—C11—C10	121.57 (16)	H19A—C19—H19C	109.5
C12—C11—H11	119.2	H19B—C19—H19C	109.5
C10—C11—H11	119.2	C1—O1—C16	119.85 (15)
O4—C12—C11	124.71 (16)	C3—O2—C17	118.05 (17)
O4—C12—C13	115.65 (15)	C12—O4—C18	117.21 (14)
C11—C12—C13	119.64 (17)	C13—O5—C19	117.86 (18)
O5—C13—C14	125.41 (17)		
			
O1—C1—C2—C3	−177.22 (16)	C15—C10—C11—C12	−0.2 (2)
C6—C1—C2—C3	−0.7 (3)	C9—C10—C11—C12	178.26 (15)
C1—C2—C3—O2	178.59 (16)	C10—C11—C12—O4	−179.63 (15)
C1—C2—C3—C4	−0.7 (3)	C10—C11—C12—C13	−0.4 (2)
O2—C3—C4—C5	−177.74 (17)	O4—C12—C13—O5	−0.4 (2)
C2—C3—C4—C5	1.6 (3)	C11—C12—C13—O5	−179.68 (14)
C3—C4—C5—C6	−1.2 (3)	O4—C12—C13—C14	−179.86 (15)
O1—C1—C6—C5	177.78 (15)	C11—C12—C13—C14	0.9 (3)
C2—C1—C6—C5	1.1 (2)	O5—C13—C14—C15	179.90 (17)
O1—C1—C6—C7	0.2 (2)	C12—C13—C14—C15	−0.7 (3)
C2—C1—C6—C7	−176.47 (15)	C13—C14—C15—C10	0.1 (3)
C4—C5—C6—C1	−0.2 (3)	C11—C10—C15—C14	0.3 (3)
C4—C5—C6—C7	177.62 (16)	C9—C10—C15—C14	−178.11 (16)
C1—C6—C7—O3	150.16 (17)	C2—C1—O1—C16	−12.8 (3)
C5—C6—C7—O3	−27.4 (2)	C6—C1—O1—C16	170.51 (17)
C1—C6—C7—C8	−31.7 (2)	C2—C3—O2—C17	−3.2 (3)
C5—C6—C7—C8	150.79 (16)	C4—C3—O2—C17	176.09 (18)
O3—C7—C8—C9	−1.9 (3)	C11—C12—O4—C18	2.6 (2)
C6—C7—C8—C9	179.96 (15)	C13—C12—O4—C18	−176.60 (15)
C7—C8—C9—C10	−176.58 (15)	C14—C13—O5—C19	−3.6 (3)
C8—C9—C10—C15	178.36 (17)	C12—C13—O5—C19	176.98 (16)
C8—C9—C10—C11	0.0 (3)		

### Hydrogen-bond geometry (Å, °)

**Table:**

*D*—H···*A*	*D*—H	H···*A*	*D*···*A*	*D*—H···*A*
C8—H8···O1	0.96 (2)	2.29 (2)	2.813 (3)	113.6 (15)
C18—H18B···O3^i^	0.96	2.46	3.253 (3)	140

Symmetry codes: (i) *x*, −*y*+1/2, *z*+1/2.
